# Plaque histology and myocardial disease in sudden coronary death: the Fingesture study

**DOI:** 10.1093/eurheartj/ehac533

**Published:** 2022-09-29

**Authors:** Lauri Holmström, Samuli Juntunen, Juha Vähätalo, Lasse Pakanen, Kari Kaikkonen, Anette Haukilahti, Tuomas Kenttä, Jani Tikkanen, Ville Viitasalo, Juha Perkiömäki, Heikki Huikuri, Robert J Myerburg, Juhani Junttila

**Affiliations:** Research Unit of Internal Medicine, Medical Research Center Oulu, University of Oulu and Oulu University Hospital, PO Box 5000, 90014 Oulu, Finland; Research Unit of Internal Medicine, Medical Research Center Oulu, University of Oulu and Oulu University Hospital, PO Box 5000, 90014 Oulu, Finland; Research Unit of Internal Medicine, Medical Research Center Oulu, University of Oulu and Oulu University Hospital, PO Box 5000, 90014 Oulu, Finland; Forensic Medicine Unit, Finnish Institute for Health and Welfare (THL), PO Box 310, 90101 Oulu, Finland; Department of Forensic Medicine, Research Unit of Internal Medicine, Medical Research Center Oulu, University of Oulu, PO Box 5000, 90014 Oulu, Finland; Research Unit of Internal Medicine, Medical Research Center Oulu, University of Oulu and Oulu University Hospital, PO Box 5000, 90014 Oulu, Finland; Research Unit of Internal Medicine, Medical Research Center Oulu, University of Oulu and Oulu University Hospital, PO Box 5000, 90014 Oulu, Finland; Research Unit of Internal Medicine, Medical Research Center Oulu, University of Oulu and Oulu University Hospital, PO Box 5000, 90014 Oulu, Finland; Research Unit of Internal Medicine, Medical Research Center Oulu, University of Oulu and Oulu University Hospital, PO Box 5000, 90014 Oulu, Finland; Research Unit of Internal Medicine, Medical Research Center Oulu, University of Oulu and Oulu University Hospital, PO Box 5000, 90014 Oulu, Finland; Research Unit of Internal Medicine, Medical Research Center Oulu, University of Oulu and Oulu University Hospital, PO Box 5000, 90014 Oulu, Finland; Research Unit of Internal Medicine, Medical Research Center Oulu, University of Oulu and Oulu University Hospital, PO Box 5000, 90014 Oulu, Finland; Department of Medicine, División of Cardiology, University of Miami, Miller School of Medicine, Miami, FL 33136, USA; Research Unit of Internal Medicine, Medical Research Center Oulu, University of Oulu and Oulu University Hospital, PO Box 5000, 90014 Oulu, Finland

**Keywords:** Sudden cardiac death, Coronary artery disease, Acute plaque complication, Cardiac hypertrophy, Myocardial fibrosis

## Abstract

**Aims:**

At least 50% of deaths due to coronary artery disease (CAD) are sudden cardiac deaths (SCDs), but the role of acute plaque complications on the incidence of sudden death in CAD is somewhat unclear. The present study aimed to investigate plaque histology and concomitant myocardial disease in sudden coronary death.

**Methods and results:**

The study population is derived from the Fingesture study, which has collected data from 5869 consecutive autopsy-verified SCD victims in Northern Finland (population ≈600 000) between 1998 and 2017. In this substudy, histological examination of culprit lesions was performed in 600 SCD victims whose death was due to CAD. Determination of the cause of death was based on the combination of medical records, police reports, and autopsy data. Plaque histology was classified as either (i) plaque rupture or erosion, (ii) intraplaque haemorrhage, or (iii) stable plaque. The mean age of the study subjects was 64.9 ± 11.2 years, and 82% were male. Twenty-four per cent had plaque rupture or plaque erosion, 24% had an intraplaque haemorrhage, and 52% had a stable plaque. Myocardial hypertrophy was present in 78% and myocardial fibrosis in 93% of victims. The presence of myocardial hypertrophy or fibrosis was not associated with specific plaque histology.

**Conclusion:**

Less than half of sudden deaths due to CAD had evidence of acute plaque complication, an observation which is contrary to historical perceptions. The prevalence of concomitant myocardial disease was high and independent of associated plaque morphology.

## Structured Graphical Abstract

**Structured Graphical Abstract:**
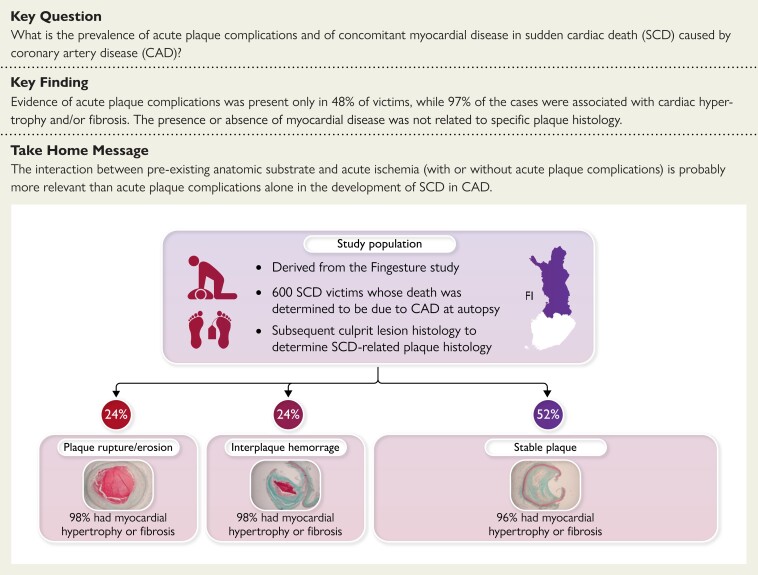
Less than half of sudden deaths due to coronary artery disease had evidence of acute plaque complications and the prevalence of concomitant myocardial disease was high. CAD, coronary artery disease; SCD, sudden cardiac death.

**See the editorial comment for this article ‘Ischaemic myocardial fibrosis is the villain of sudden coronary death’, by Gaetano Thiene, https://doi.org/10.1093/eurheartj/ehac571.**

## Introduction

Sudden cardiac death (SCD) accounts for ∼50% of total cardiovascular mortality and 10%–20% of all deaths.^1^ Most of the SCDs (70%–80%) are caused by coronary artery disease (CAD), whereas non-ischaemic myocardial diseases account for approximately one-fifth of the cases, and the minority is caused by other cardiac conditions (e.g. primary arrhythmia syndromes, valve diseases, or myocarditis).^2,3^ Although treatments of CAD have improved significantly and led to declined age-adjusted mortality^4^ in the current era, the overall incidence of SCD has remained relatively stable.^5^

The common perception is that CAD-associated SCD is divided into two pathophysiological mechanisms: acute plaque complications causing acute coronary syndrome (ACS) and SCD, and prior myocardial infarction (MI) scar as a substrate for re-entry arrhythmias.^2^ Early studies from the late 1980s and 1990s reported a high prevalence of acute plaque complications in sudden death due to CAD.^6,7^ However, given that the high prevalence was partly due to a subset of SCDs occurring during physical activity,^6^ recent progress in CAD management, and changes in patient characteristics, the previous results may not reflect the overall impact of acute plaque complications on the occurrence of SCD in the current era. In addition, recent randomized trials have found no benefit of early angiography among sudden cardiac arrest (SCA) victims without ST-segment elevation,^8,9^ which may suggest that the role of acute plaque complications on the overall occurrence of SCA is not as robust as previously hypothesized.

Along with CAD, left ventricular hypertrophy (LVH) and myocardial fibrosis are common findings in autopsy following SCD. Myocardial disease may act as an anatomic substrate for SCD and predispose to and/or maintain potential fatal ventricular arrhythmias via the re-entry mechanism.^2^ The most common causes for hypertrophic and fibrotic myocardial diseases are untreated hypertension, obesity, or valve diseases, but some cases may be attributable to an inherited predisposition based upon genetic variants.^10^

Considering the gaps in the current knowledge of the anatomic substrates for SCD in CAD, our aim in this study was to investigate the prevalence of plaque complications, in association with concomitant myocardial disease in SCD caused by CAD.

## Methods

### The Fingesture study

The study population is derived from The Finnish Genetic Study of Arrhythmic Events (Fingesture) which has gathered medical records and medicolegal autopsy data from 5869 consecutive SCD victims since 1998 from the Oulu University Hospital District (defined geographical area in northern Finland, population ≈600 000). The detailed study protocol has been previously described and a brief description follows.^3,10–12^

Finnish law requires a medicolegal autopsy to be performed if (i) death is not due to a known disease, (ii) the victim has not been treated during his/her last illness, or (iii) the death is otherwise unexpected. Accordingly, all victims of sudden and unexpected death undergo meticulous post-mortem investigations in Finland. Because of this, Finland has the highest autopsy rate following sudden death in Western societies.^13,14^

The Fingesture study included all victims of sudden death that were determined to be due to cardiac disease. Non-cardiac causes (e.g. pulmonary embolism, aortic rupture, cerebrovascular event, trauma, and intoxication) were excluded from the Fingesture study. Determination of the cause of death was based on the combination of data in medical records, police reports, and autopsy data. Additionally, a questionnaire was sent to the next of kin of the deceased for research purposes. All SCD victims in the Fingesture underwent a medicolegal autopsy in the Finnish Institute for Health and Welfare, Oulu, Finland, or at the Department of Forensic Medicine, University of Oulu, Oulu, Finland. Autopsies were performed by experienced forensic pathologists, each performing over 100 autopsies/year, using a uniform study protocol and contemporary guidelines for diagnosing the cause of death. Causes of SCD in the Fingesture study have been reported previously.^3,15^

### Subjects in the present study

The present substudy included only SCD victims whose death was considered ischaemia-associated in the presence of CAD. Sudden cardiac death victims with evidence of non-ischaemic myocardial disease, a mechanical complication of MI such as rupture of the myocardium and/or tamponade, or any other cardiac cause of SCD not considered as ischaemia-induced were excluded. Coronary artery disease–related SCD was defined as histological culprit lesion findings of acute thrombus, plaque rupture or erosion, intraplaque haemorrhage, or critical coronary stenosis (>75%) in a main coronary artery and no other cause of sudden death (e.g. cardiomyopathy, valve disease, aortic rupture, pulmonary embolism, stroke, and intoxication). The routine autopsy protocol also included myocardial dissection, valve examination, heart weight measurement, and assessment of myocardial fibrosis based on macroscopic and histological analysis of tissue samples from the heart muscle. Quantification of myocardial fibrosis was based on forensic pathologists’ visual assessment and was categorized into four groups: (i) no fibrosis, (ii) scattered mild fibrosis, (iii) moderate patchy fibrosis, and (iv) substantial fibrosis.

**Figure 1:**
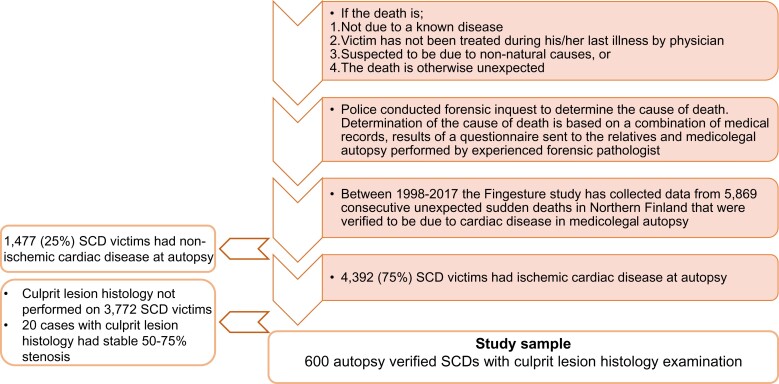
Description of post-mortem procedure in Finland and study subject selection. SCD, sudden cardiac death.

**Figure 2:**
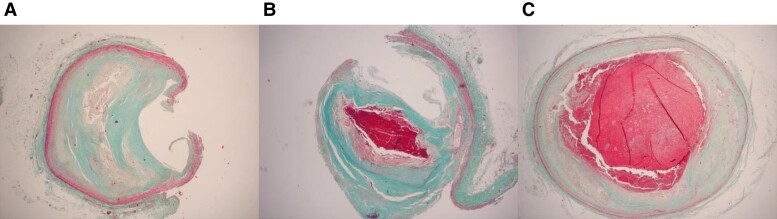
Examples of various culprit lesion morphologies in histological analysis with the Masson-Trichrome stain: (*A*) stable plaque, (*B*) intraplaque haemorrhage, and (*C*) plaque rupture with thrombosis.

This substudy population included CAD-associated SCD victims who had detailed histological data on the culprit lesion (*Figure 1*). Autopsies in the Fingesture study are randomly performed by a few forensic pathologists, of which one performed a detailed histological analysis of the culprit lesion, in addition to routine autopsy protocol. Consequently, the histological information of the culprit lesion was available in 600 randomly selected SCD victims, and all the autopsies and histological examinations of the coronary arteries were performed by the same experienced forensic pathologist. The culprit lesion was histologically defined as a plaque with an acute thrombus, or in the absence of thrombus or plaque rupture, the arterial segment with the greatest narrowing or deep haemorrhage into the plaque without plaque disruption. Based on the histological examination, the culprit lesion was categorized into one of three classes: (i) plaque rupture or plaque erosion, (ii) intraplaque haemorrhage, and (iii) stable plaque (*Figure 2*). The severity of CAD was determined by the number of the large epicardial coronary arteries affected (left main, and/or left anterior descending, and/or left circumflex, and/or right coronary artery) which had significant stenosis (>75% occlusion). The presence of old and fresh MI was verified by histological examination of myocardial samples. Cardiac hypertrophy was determined by measured heart weight at autopsy, defined as ≥420 g in men and ≥350 g in women, based on previously defined normal heart weight values in the Finnish autopsy study of deaths from external causes.^16^

Information regarding physical activity preceding death was gathered from police reports, death certificates, and information from witnesses. Metabolic equivalents of various activities were estimated according to a study by Jetté *et al*.,^17^ and SCD was considered to associate with physical exercise if the preceding activity was equivalent to ≥4 metabolic equivalents. This definition included a broad spectrum of activities ranging from everyday chores to vigorous exertion. If the victim was sitting, sleeping, or lying down at the time of death, SCD was considered to occur at rest. Based on the preceding activity, SCD victims were classified as either (i) SCD during exercise, (ii) SCD <1 h after exercise, (iii) SCD during mild exercise, and (iv) SCD at rest. Body surface area was calculated using Mosteller’s formula. We were able to collect prior ECG recordings (unrelated to the SCD event) from 51 study subjects. Definitions of ECG characteristics are described earlier.^3,15^

### Statistical methods

All continuous variables are presented as mean ± standard deviation. We used analysis of variances (ANOVA) and *χ*^2^ test to estimate the statistical significance of continuous and categorical variable distributions between the study groups of interest, respectively. If there was a statistically significant difference between the three study groups, further *post hoc* analyses were performed with Bonferroni correction. All variables with *P* < 0.10 in *χ*^2^/ANOVA analyses were included in the multivariable logistic regression models. All analyses were performed with Statistical Package for Social Studies (version 28.0.0.0). All reported *P*-values are two-sided and values <0.05 were considered statistically significant.

The study complies with the Declaration of Helsinki, and the ethics committee of the University of Oulu approved the study. The Finnish Institute for Health and Welfare, and Regional State Administrative Agency of Northern Finland approved the review of medicolegal autopsy data by the investigators. Consent from next of kin was waived by the ethics committee since according to the Finnish law, medicolegal autopsy does not require a consent.

## Results

### Subject characteristics and plaque morphology

**Figure 3:**
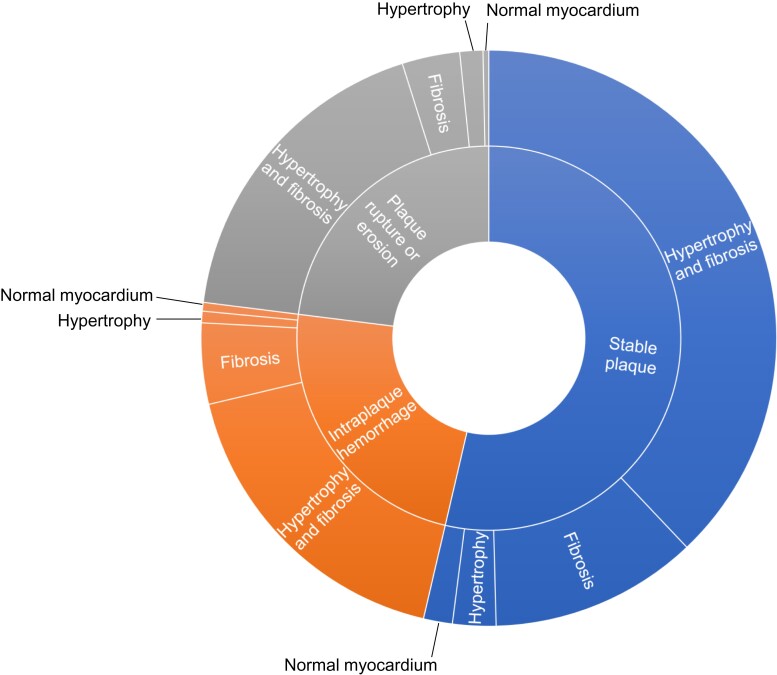
Proportions of culprit lesion morphologies and type of myocardial disease according to plaque morphology.

The mean age of the study subjects was 64.9 ± 11.2 years, and 82% were male. The mean body mass index was 27.2 ± 4.8 kg/m^2^. Forty-eight per cent had been smokers. Culprit lesion morphology was a plaque rupture or erosion in 23.7%, intraplaque haemorrhage in 24.0%, and stable plaque in 52.3% (*Figure 3*). Those with plaque rupture or erosion were younger (62.6 ± 11.8 years) than those with intraplaque haemorrhage (66.1 ± 11.1 years; *P* = 0.018). Plaque rupture or erosion was also associated with lower prevalence of previous MI (7.2% vs. 16.9 vs. 15.6%; *P* = 0.03) than intraplaque haemorrhage or stable plaque, respectively. There were no statistically significant associations between the prevalence of one-, two-, or three-vessel disease and culprit lesion morphology. There were no statistically significant sex-based differences in the proportion of plaque rupture/erosion (25.0% in men vs. 17.6% in women), intraplaque haemorrhage (23.8% in men vs. 25.0% in female), or stable plaque (51.2% in men vs. 57.4% in female) (*P* = 0.25). However, this may be due to the low number of female SCD victims and insufficient statistical power. Study subject characteristics according to plaque morphology are presented in *Table 1*. Additional clinical characteristics are presented in Supplementary material online, *Table S1*, and electrocardiographic characteristics are presented in Supplementary material online, *Table S2*. In a multivariable logistic regression model, acute infarct scar at autopsy and SCD within 1 h after exercise were independently associated with plaque rupture/erosion (see Supplementary material online, *Table S3*).

**Table 1:** Demographic and autopsy findings of the study subjects according to plaque morphology

	All (*n* = 600, 100%)	Plaque rupture or erosion (*n* = 142, 23.7%)	Intraplaque haemorrhage (*n* = 144, 24.0%)	Stable plaque or no acute lesion (*n* = 314, 52.3%)	*P*-value (*post hoc*)
Age, years	64.9 ± 11.2	62.6 ± 11.8	66.1 ± 11.1	65.3 ± 10.8	**0.018** *
Male sex	492/600 (82.0%)	123/142 (86.6%)	117/144 (81.3%)	252/314 (80.3%)	0.25
BMI, kg/m^2^	27.2 ± 4.8	27.7 ± 4.3	27.6 ± 5.0	26.7 ± 4.8	0.098
Total heart weight, g	481.5 ± 107.5	490.8 ± 95.9	494.1 ± 111.2	471.5 ± 110.0	0.056
BMI-adjusted, g/kg/m^2^	17.9 ± 3.7	17.9 ± 3.4	18.2 ± 3.8	17.7 ± 3.7	0.405
BSA-adjusted, g/m^2^	248.3 ± 42.4	247.2 ± 41.1	255.0 ± 45.0	245.7 ± 41.6	0.10
**Degree of fibrosis**					0.848
Any fibrosis	559/600 (93.2%)	132/142 (93.0%)	137/144 (95.1%)	290/314 (92.4%)	NS
Substantial	67/600 (11.2%)	12/142 (8.5%)	18/144 (12.5%)	37/314 (11.8%)	NS
Patchy	405/600 (67.5%)	98/142 (69.0%)	99/144 (68.8%)	208/314 (66.2%)	NS
Scattered mild	87/600 (14.5%)	22/142 (15.5%)	20/144 (13.9%)	45/314 (14.3%)	NS
None	41/600 (6.8%)	10/142 (7.0%)	7/144 (4.9%)	24/314 (7.6%)	NS
**Circumstances during SCD**					**0.003**
SCD during exercise	108/593 (18.2%)	17/139 (12.2%)	30/144 (20.8%)	61/310 (19.7%)	NS
SCD within 1 h after exercise	29/593 (4.9%)	14/139 (10.1%)	9/144 (6.3%)	6/310 (1.9%)	***
SCD at rest	162/593 (27.3%)	40/139 (28.8%)	42/144 (29.2%)	80/310 (25.8%)	NS
Mild activity	294/593 (49.6%)	68/139 (48.9%)	63/144 (43.8%)	163/310 (52.6%)	NS
**>75% stenosis in main artery**					0.549
One-vessel disease	116/545 (21.3%)	20/130 (15.4%)	26/136 (19.1%)	70/279 (25.1%)	NS
Two-vessel disease	131/545 (24.0%)	31/130 (23.8%)	27/136 (19.9%)	73/279 (26.2%)	NS
Three-vessel disease	249/545 (45.7%)	48/130 (36.9%)	65/136 (47.8%)	136/279 (48.7%)	NS
Heart weight over reference values	464/598 (77.6%)	120/142 (84.5%)	113/144 (78.5%)	231/312 (74.0%)	0.050
Acute infarct scar at autopsy	331/592 (55.9%)	103/140 (73.6%)	85/140 (60.7%)	143/312 (45.8%)	**<0.001** **,***
Old infarct scar at autopsy	344/595 (57.8%)	74/140 (52.9%)	94/144 (65.3%)	176/311 (56.6%)	0.086
Fibrosis and heart weight over reference values	439/598 (73.4%)	112/142 (78.9%)	109/144 (75.7%)	218/312 (69.9%)	0.101
Normal myocardium	16/600 (2.7%)	2/142 (1.4%)	3/144 (2.1%)	11/314 (3.5%)	0.386

If the omnibus *P*-value was <0.05, *post hoc* analysis with Bonferroni correction was applied. Only statistically significant pairwise comparison results are marked in the footnotes. The bold values represent statistically significant (*P* < 0.05) comparisons.

BMI, body mass index; BSA, body surface area; MI, myocardial infarction; SCD, sudden cardiac death.

* Significance (*P* < 0.05) between intraplaque haemorrhage and plaque rupture or erosion groups.

** Significance (*P* < 0.05) between stable plaque and intraplaque haemorrhage groups.

*** Significance (*P* < 0.05) between stable plaque and plaque rupture or erosion groups.

### Myocardial disease

Heart weight was greater than the normal value in 78% of subjects, and 93% of subjects had increased fibrosis compared with the same age group without cardiac disease. Only 2.7% of the SCD victims had heart weight in normal values and no fibrosis. Healed MI scars were present in 58%, and 56% had signs of acute MI at autopsy. Acute MI was more common among those with plaque rupture or erosion (74%) in comparison to intraplaque haemorrhage (61%) or stable plaque (46%; *P* < 0.001). There were no statistically significant differences in the prevalence of hypertrophy, fibrosis, or old infarct scar based upon plaque morphology.

### Physical exercise

Eighteen per cent of the SCDs occurred during physical exercise, whereas 5% occurred <1 h after exercise, 50% during mild activity, and 27% at rest. There was no statistically significant association between plaque histology and SCD during exercise. Plaque rupture/erosion occurred more often within 1 h after exercise than intraplaque haemorrhage (10.1% vs. 1.9%; *P* = 0.003). Nevertheless, there was no statistically significant difference in the occurrence of SCD during mild activity or at rest among those with plaque rupture or erosion in comparison to intraplaque haemorrhage and stable plaque.

## Discussion

In this autopsy-based study of 600 SCD victims whose death was attributable to CAD, there were two main findings: (i) evidence of acute plaque changes was present only in 48% of victims and (ii) 97% of the cases were associated with cardiac hypertrophy and/or fibrosis. The presence or absence of myocardial disease was not related to specific plaque histology (*Structured Graphical Abstract*). In addition, those whose death occurred after physical exercise more commonly had plaque rupture or erosion, and those with plaque rupture/erosion were somewhat younger on average and had more commonly signs of recent MI at autopsy.

The common perception is that ischaemic SCA associates with either prior MI scar-related ventricular tachycardia via a re-entrant mechanism or acute ischaemia due to plaque rupture and thrombogenic total occlusion of the coronary artery.^2^ Our results somewhat challenge the traditional, dualistic paradigm by introducing a spectrum of possibilities for ischaemic SCA. Coronary pathology can include an acute plaque rupture, intraplaque haemorrhage, or stable plaque, which results in lethal arrhythmogenic spiral in unison with significant myocardial disease. The myocardial disease might not specifically be due to prior infarct scar but can also be due to interplay of prolonged ischaemia and hypertrophic remodelling. The role of myocardial disease seems to be pivotal since 97% of SCD subjects had significant myocardial disease in our study.

In contrast to our results, Burke *et al*.^6^ reported that among 116 SCDs occurring at rest and 25 during physical activity or emotional stress, plaque rupture was more common in SCDs due to physical activity (68%) than in SCD at rest (23%). They did not, however, separate SCDs occurring <1 h after exertion/stress from SCDs occurring during exertion/stress, and it is unclear whether the association between plaque rupture and exertion was due to SCDs occurring during or <1 h after exertion. Hence, their results are not necessarily contradictory to ours. Nonetheless, these results should be interpreted with caution since a notable proportion of SCDs in both studies were unwitnessed and determining the level of exertion prior to unwitnessed SCD may have major limitations. In their earlier study, Burke *et al*.^18^ reported that abnormal serum cholesterol levels and smoking were associated with plaque rupture and acute thrombosis. In our study, we had no data about serum cholesterol levels to replicate these results, and the overall prevalence of smoking was lower, which may mitigate associations with plaque morphologies.

Previous studies have reported a high prevalence of plaque complications among SCD victims,^6,7^ which is the basis for the concept that vulnerable plaques gained a lot of interest in the research field, in the anticipation that identifying such lesions would enable effective preventive intervention. However, this hypothesis was based on limited data and somewhat false assumptions. Subsequent studies demonstrated that most culprit lesions were nonobstructive prior to the event^19,20^ and that asymptomatic atherosclerotic lesions and plaque complications are common in subjects without clinical CAD.^21,22^ In addition, ‘high-risk’ plaques represent merely a surrogate marker for total CAD severity, which is a more important risk factor for ACS.^23,24^ The focus on ACS prediction has subsequently transitioned from vulnerable plaques to overall atherosclerotic disease burden, plaque progression, and the additional factor of individual response.^25^ Similar to the previous hypothesis of vulnerable plaques and ACS, direct relationship between acute plaque complications and SCA has its limitations due to the fact that other factors related to SCA substrate are ignored. Given that the rate of prehospital SCA in ST-elevation MI (STEMI) was recently evaluated to be only 5%,^26^ other factors are likely to explain a notable proportion of individuals’ propensity to lethal arrhythmias, especially in the early phase of MI.

Although angiography immediately following SCA due to STEMI has apparent benefits, recent randomized studies (COACT and TOMAHAWK) demonstrated no benefit from early angiography among resuscitated SCA victims without ST-segment elevation.^8,9^ Consequently, current European Society of Cardiology guidelines do not recommend immediate angiography routinely after resuscitated SCA without ST-segment elevation, while resuscitated SCAs with ST-segment elevation are recommended to undergo immediate angiography.^27^ The reasons for these observations in randomized trials are not fully clear but may be due to lesser significance of acute plaque complications on the occurrence of SCA than previously hypothesized. Indeed, clinically significant disease was present in 65% of patients in both trials, and in the COACT trial only 23% of patients with CAD had acute unstable lesion.^9^ Comparing our results to these trials may have caveats as the culprit lesion classification is not as robust in post-resuscitation angiography as in histological examination. Nonetheless, our results report a slightly higher amount of acute plaque complications, which may be due to our study design which likely captured some SCAs with ST-segment elevation as well. Altogether, both SCD victims and survivors seem to have most often stable coronary plaques.

Considering that 46% of the SCD victims without acute plaque complications in the present study had already signs of recent MI at autopsy, vasospasm and transient ischaemia may also be a significant contributor to SCD among CAD patients with pre-existing myocardial disease. Furthermore, the overall prevalence of SCDs due to CAD has declined, whereas SCDs attributable to non-ischaemic myocardial diseases, especially hypertensive myocardial disease, and primary myocardial fibrosis, have increased during the recent decades.^5^

**Figure 4:**
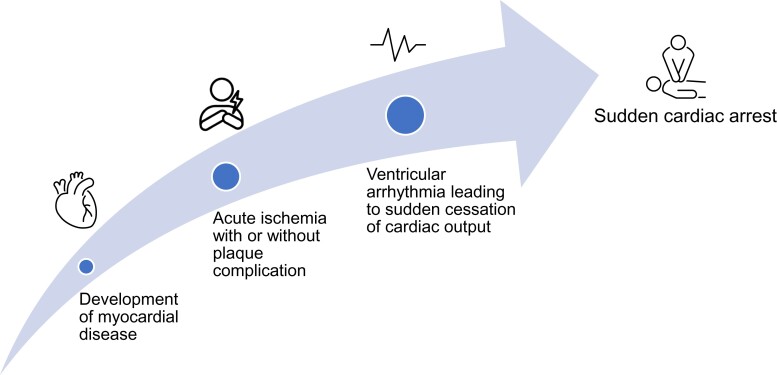
Schematic illustration of hypothesis of sudden cardiac arrest pathogenesis in coronary artery disease.

Extensive autopsy studies of various SCD victims have reported a high prevalence of cardiac hypertrophy and myocardial fibrosis, regardless of whether the cause was ischaemic or non-ischaemic.^3,15,28,29^ It is not fully clear whether hypertrophy without scarring has the same arrhythmic potential as isolated fibrosis since hypertrophy and fibrosis usually coexist. However, a recent study on CAD patients demonstrated that patients without myocardial fibrosis in cardiac magnetic resonance imaging had an excellent prognosis with regard to SCD,^30^ suggesting that myocardial fibrosis may be one of the most important determinants of the arrhythmic risk. In the Framingham study, subjects with LVH had a 2.2-fold increased risk of SCD.^31^ Our previous case–control study of SCD victims and survivors of acute MI demonstrated that LVH was associated with a three-fold risk of death.^32^ Causes of cardiac hypertrophy and fibrosis in the present study were probably diverse, but CAD and hypertension are likely to account for the majority of myocardial diseases. However, predisposition to cardiac hypertrophy may also be inherited,^33^ and previous studies have repeatedly reported that a family history of SCD increases susceptibility to SCD/ventricular fibrillation during MI and in long-term follow-up.^11,34,35^ More than one-half of the SCD victims in the present study had an old myocardial scar at autopsy, which may also be accountable for a notable proportion of SCDs even in the absence of previously diagnosed CAD.^12^ Altogether, given the prevalence of plaque complications and myocardial disease among SCD victims, interaction between pre-existing anatomic substrate and acute ischaemia (either Type 1 or Type 2) is probably more relevant than plaque complications alone in the development of life-threatening arrhythmias and SCD (*Figure 4*). As long ago as 1992, Szlachcic *et al*.^36^ demonstrated that among hypertensive patients with LVH, silent ischaemia is associated with an increased risk of ventricular arrhythmias. Left ventricular hypertrophy regression among hypertensive patients has been associated with a decreased risk of SCD,^37^ and preventive measures of SCD in CAD should also highlight the treatment of concomitant myocardial disease.

### Strength and limitations

Our study has some limitations that should be recognized. First, only a subpopulation of all SCDs due to CAD underwent coronary artery examination. Despite this, the present study includes more SCD victims with coronary artery histology examinations than any previous report. The ability to collect such a large sample of SCD victim autopsy data is due to Finnish legislation, which requires medicolegal autopsy to be performed in case of unexplained death, regardless of the victims’ age, leading to a unified investigation protocol. Study subjects were drawn from one forensic pathologist’s autopsies, which may be considered random. Secondly, histological examination was performed only for the culprit lesion, and no data are available about other less significant lesions. Additionally, we lacked data on presenting rhythms for most of the cases, and thus, we were not able to analyse the association between plaque histology and presenting rhythm. Our study population has a homogeneous ethnic background (almost 100% white Caucasian), and we did not have the statistical power to perform an analysis related to differences in ethnicity. We also lacked a significant amount of data on smoking status. We neither had information available on the exact amount nor the localization of myocardial fibrosis. Finally, the Fingesture study has gathered data about autopsied SCD victims during a long period of time, and developing management strategies for CAD may have led to temporal trends in the plaque morphologies, which were not assessed in the present study.

## Conclusions

Acute plaque complications were present in less than one-half of the SCD victims whose SCA was determined to be due to CAD. The prevalence of concomitant myocardial disease was high, with 97% of the victims having either cardiac hypertrophy or myocardial fibrosis at autopsy. Myocardial disease was present at the same prevalence regardless of the culprit plaque histology. The clinical significance of these results lies in the importance of recognizing the effect and the variable individual arrhythmogenic response to ischaemia.

## Supplementary material

Supplementary material is available at *European Heart Journal* online.

## Supplementary Material

ehac533_Supplementary_Data

## Data Availability

The data underlying this article cannot be shared publicly due to their potentially identifiable nature.
