# Supplementary material for: Plaque histology and myocardial disease in sudden coronary death: the Fingesture study
Source: Eur Heart J. 2022 Sep 29;43(47):4923–30. doi: 10.1093/eurheartj/ehac533 (PMC9748531; doi:10.1093/eurheartj/ehac533)
Supplement: ehac533_Supplementary_Data [file ehac533_supplementary_data.zip › CAD_SCD_SupplementTable1.docx]

|  | All (n=600, 100%) | Plaque rupture or erosion (n=142, 23.7%) | Intraplaque hemorrhage  (n=144, 24%) | Stable plaque or no acute lesion (n=314, 52.3%) | p value |
| --- | --- | --- | --- | --- | --- |
| Pre-SCD clinical history |  |  |  |  |  |
| Previous MI | 82/588 (13.9%) | 10/139 (7.2%) | 24/142 (16.9%) | 48/307 (15.6%) | **0.031 b, c** |
| Diabetes | 98/581 (16.9%) | 16/136 (11.8%) | 23/141 (16.3%) | 59/304 (19.4%) | 0.137 |
| Hypertonia | 270/581 (46.5%) | 71/138 (51.4%) | 64/141 (45.4%) | 135/302 (44.7%) | 0.401 |
| Angina Pectoris | 265/579 (45.7%) | 57/136 (41.9%) | 65/139 (46.8%) | 143/304 (47.0%) | 0.591 |
| TIA or Ischemic Stroke | 36/546 (6.6%) | 8/130 (6.2%) | 8/134 (6.0%) | 20/282 (7.1%) | 0.899 |
| Parent’s SCD | 56/286 (19.6%) | 14/74 (18.9%) | 14/65 (21.5%) | 28/147 (19.0%) | 0.922 |
| 1st degree family member’s SCD | 83/241 (34.4%) | 20/60 (33.3%) | 23/59 (39.0%) | 40/122 (32.8%) | 0.708 |
| Smoker | 172/358 (48.0%) | 47/88 (53.4%) | 36/89 (40.4%) | 89/181 (49.2%) | 0.203 |
| Medications |  |  |  |  |  |
| Cholesterol medication | 134/504 (26.6%) | 30/119 (25.2%) | 28/126 (22.2%) | 76/259 (29.3%) | 0.312 |
| Beta Blocker | 110/417 (26.4%) | 26/107 (24.3%) | 29/102 (28.4%) | 55/208 (26.4%) | 0.802 |
| Nitrate (long- or short-acting) | 97/428 (22.7%) | 25/108 (23.1%) | 24/103 (23.3%) | 48/217 (22.1%) | 0.965 |
| Aspirin | 63/418 (15.1%) | 15/108 (13.9%) | 13/102 (12.7%) | 35/208 (16.8%) | 0.598 |
| Diuretic | 61/416 (14.7%) | 16/106 (15.1%) | 15/102 (14.7%) | 30/208 (14.4%) | 0.987 |
| Warfarin | 26/421 (6.2%) | 3/106 (2.8%) | 8/103 (7.8%) | 15/212 (7.1%) | 0.266 |
| ACE-I or ARB | 73/416 (17.5%) | 18/106 (17.0%) | 15/102 (14.7%) | 40/208 (19.2%) | 0.613 |
| Digoxin | 22/417 (5.3%) | 4/106 (3.8%) | 5/102 (4.9%) | 13/209 (6.2%) | 0.630 |
| CCB | 34/417 (8.2%) | 7/106 (6.6%) | 8/102 (7.8%) | 19/209 (9.1%) | 0.733 |

**Supplement table 1**. Pre-SCD clinical history and medications of the study subjects according to plaque morphology. If the omnibus p-value was <0.05, post hoc analysis with Bonferroni correction was applied. Only statistically significant pairwise comparison results are marked in the footnotes. a=Significance (p<0.05) between stable plaque and intraplaque hemorrhage groups, b=significance (p<0.05) between stable plaque and plaque rupture or erosion groups, c=significance (p<0.05) between intraplaque hemorrhage and plaque rupture or erosion groups. MI=myocardial infarction, TIA=transient ischemic attack, SCD=sudden cardiac death, ACE-I=angiotensin-converting enzyme inhibitor, ARB=angiotensin receptor blocker, CCB=calcium channel blocker.
