# Supplementary material for: Plaque histology and myocardial disease in sudden coronary death: the Fingesture study
Source: Eur Heart J. 2022 Sep 29;43(47):4923–30. doi: 10.1093/eurheartj/ehac533 (PMC9748531; doi:10.1093/eurheartj/ehac533)
Supplement: ehac533_Supplementary_Data [file ehac533_supplementary_data.zip › CAD_SCD_SupplementTable2.docx]

|  | All (n=51) | Plaque rupture or erosion (n=7) | Intraplaque hemorrhage (n=14) | Stable plaque or no acute lesion  (n=30) | p value |
| --- | --- | --- | --- | --- | --- |
| Heart rate, bpm | 79.4±18.4 | 76.7±13.2 | 79.2±20.1 | 80.1±19.2 | 0.911 |
| Atrial fibrillation | 6/51 (11.8%) | 1/7 (14.3%) | 2/14 (14.3%) | 3/30 (10.0%) | 0.896 |
| QRS duration, ms | 91.7±14.1 | 94.1±12.4 | 88.9±14.4 | 92.4±14.6 | 0.673 |
| PR interval, ms | 161.0±33.9 | 164.3±24.5 | 150.0±36.6 | 165.3±34.3 | 0.370 |
| QTc duration, ms | 442.1±36.8 | 430.3±27.3 | 442.0±39.8 | 444.8±37.8 | 0.653 |
| Sokolow-Lyon index, mV | 2.2±1.0 | 2.6±0.5 | 2.0±0.8 | 2.2±1.1 | 0.501 |
| Pathologic Q waves | 7/51 (13.7%) | 0/7 (0%) | 2/14 (14.3%) | 5/30 (16.7%) | 0.639 |
| T-wave inversions | 11/51 (21.6%) | 1/7 (14.3%) | 4/14 (28.6%) | 6/30 (20.0%) | 0.796 |
| QRS fragmentation | 31/51 (60.8%) | 6/7 (85.7%) | 9/14 (64.3%) | 16/30 (53.3%) | 0.333 |
| Early repolarization | 15/51 (29.4%) | 4/7 (57.1%) | 2/14 (14.3%) | 9/30 (30.0%) | 0.158 |

**Supplement table 2.** Electrocardiographic characteristics. bpm=beats per minute.
