# Supplementary material for: Plaque histology and myocardial disease in sudden coronary death: the Fingesture study
Source: Eur Heart J. 2022 Sep 29;43(47):4923–30. doi: 10.1093/eurheartj/ehac533 (PMC9748531; doi:10.1093/eurheartj/ehac533)
Supplement: ehac533_Supplementary_Data [file ehac533_supplementary_data.zip › CAD_SCD_SupplementTable3.docx]

|  | **Plaque rupture/erosion** | | **Intraplaque hemorrhage** | | **Stable plaque** | |
| --- | --- | --- | --- | --- | --- | --- |
| **Covariate** | OR (95% CI) | p value | OR (95% CI) | p value | OR (95% CI) | p value |
| Age | 0.98 (0.96-1.00) | 0.06 | 1.02 (1.00-1.04) | 0.07 | 1.00 (0.98-1.02) | 0.98 |
| Female gender | 1.01 (0.49-2.07) | 0.99 | 1.22 (0.63-2.39) | 0.56 | 0.96 (0.53-1.72) | 0.88 |
| BMI | 0.98 (0.93-1.04) | 0.51 | 1.03 (0.98-1.09) | 0.26 | 0.99 (0.95-1.04) | 0.71 |
| Total heart weight | 1.00 (1.00-1.01) | 0.33 | 1.00 (1.00-1.00) | 0.37 | 1.00 (1.00-1.00) | 0.16 |
| Previous myocardial infarction | 0.47 (0.21-1.09) | 0.08 | 1.16 (0.63-2.12) | 0.64 | 1.25 (0.71-2.19) | 0.44 |
| Old infarct scar at autopsy | 0.71 (0.44-1.14) | 0.15 | 1.57 (0.98-2.53) | 0.06 | 0.97 (0.65-1.44) | 0.86 |
| Acute infarct scar at autopsy | 3.71 (2.07-6.63) | <0.001 | 1.19 (0.74-1.90) | 0.48 | 0.42 (0.28-0.63) | <0.001 |
| Heart weight over reference value | 1.62 (0.76-3.45) | 0.21 | 0.79 (0.40-1.57) | 0.51 | 1.01 (0.56-1.83) | 0.98 |
| SCD within 1h after exercise | 3.66 (1.28-10.41) | 0.02 | 1.31 (0.49-3.54) | 0.59 | 0.27 (0.10-0.77) | 0.01 |

**Supplement table 3.** Results from three different multivariable logistic regression models predicting each plaque morphology. All variables with p<0.10 in χ^2^/ANOVA -analyses were included in the model and all models included the same covariates. SCD=sudden cardiac death.
